# Protein mannosylation in actinobacteria an enigmatic post-translational modification

**DOI:** 10.1039/d5cb00270b

**Published:** 2025-12-05

**Authors:** Cameron B. King, Warren W. Wakarchuk

**Affiliations:** a University of Alberta, Department of Biological Sciences Edmonton Alberta Canada T6G 2E9 wakarchu@ualberta.ca

## Abstract

Protein glycosylation is a very common post-translational modification seen in all branches of biology. The functional roles for protein glycosylation are many and varied, essential in eukaryotes but seemingly dispensable in bacteria. One group of bacteria where protein glycosylation has been looked at for at least 50 years are the actinobacteria, a large and diverse group of bacteria which include well know pathogens like *Mycobacteria tuberculosis*, *Corynebacterium diphtheriae*, and well know species important in biotechnology like *Streptomyces lividans* and *Corynebacterium glutamicum.* Actinobacterial protein glycosylation is a form of protein *O*-mannosylation which is found widely in eukaryotes from single celled yeast to complex multicellular organisms but is much less understood at the functional level. Very few direct roles for protein *O*-mannosylation have been described in the literature. This review examines newer findings from the actinobacterial research literature which with the help of glycoprotein models suggests how the glycans might play a role in actinobacterial growth and physiology.

## Abstract 2

Protein glycosylation is a very common post-translational modification seen in all branches of biology.

## Graphical abstract

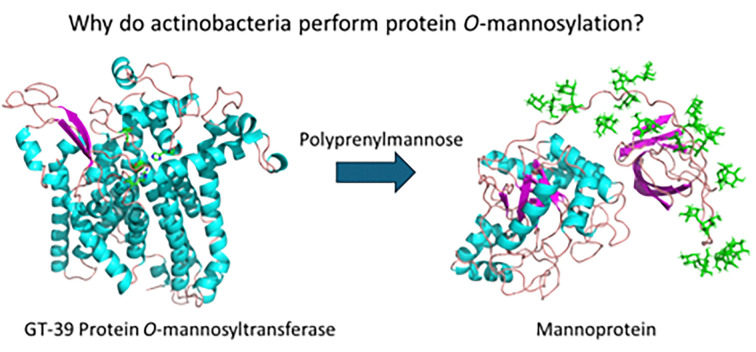

## Introduction

**Fig. 1:**
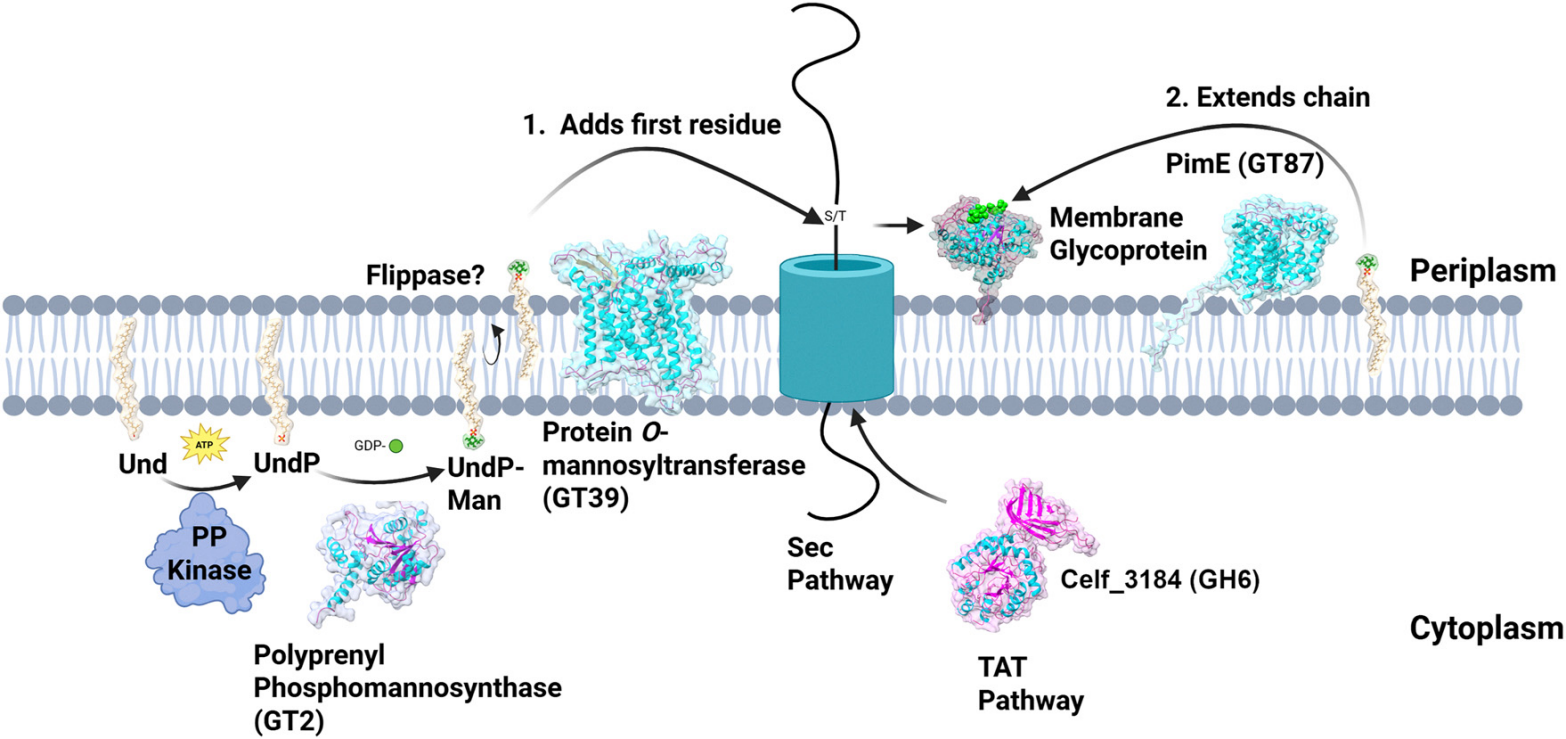
Predicted schematic of bacterial protein *O*-mannosylation pathway. We have used AlphaFold predicted structures of *Corynebacterium glutamicum* ATCC13032, *Mycobacterium smegmatis* and *Cellulomonas fimi* orthologues to illustrate proteins involved. The transmembrane GT-39 glycosylatransferase (From *C. glutamicum*, protein *O*-mannosyltransferase, UniprotID Q8NRZ6) catalyzes the transfer of a single β-d-mannose residue from a lipid donor, polyprenol phosphomannose (UndP-Man). Synthesis of the lipid donor occurs in the cytoplasm through successive reactions catalyzed by a polyprenol kinase on a C45–C75 polyprenol lipid (usually undecaprenol – Und) then a GT-2 family mannosyltransferase (shown here is the *C. glutamicum* polyprenol-phosphate β-d-mannosyltransferase, UniprotID Q8NQF7) on the resulting polyprenol phosphate (UndP). Once synthesized the lipid donor, UndP-Man, is flipped to face the periplasm by a hypothesized flippase enzyme where the mannose is transferred to a serine or threonine of a Sec or TAT secreted protein (from *Cellulomonas fim*i, Celf_3184 UniprotID Q8NLT5) in the periplasm/at the membrane surface. Additionally in the periplasm, the GT-87 enzyme PimE (From *M. smegmatis* UniprotID A0R2K8) extends the chain of mannose residues beyond the first on mannoproteins also using UndP-Man as a donor.

Protein *O*-mannosylation (POM) is a form of O-linked glycosylation (Fig. 1) which is commonly found in eukaryotic organisms from single celled yeast through to humans where it serves an essential function (reviewed in ref. 1). According to the Carbohydrate Active Enzymes Data Base (CAZY.org), the key enzyme, protein *O*-mannosyltransferase from glycosyltransferase family GT-39, is also found in ∼4000 species of bacteria, the vast majority of which are actinobacteria. In contrast to the eukaryotic systems, the actinobacterial system has been much less studied despite being so widespread in this diverse actinobacterial bacterial family.

The study of bacterial POM started at least 50 years ago with the discovery that a lipid-linked donor was required for glycoprotein synthesis in *Mycobacterium smegmatis*.^2^ Since the early discovery of these *O*-mannosylated proteins, many glycoproteins from various *Mycobacterium tuberculosis* (MTB) samples have subsequently been reported. However, it was not until 1995 when the covalent linkage of mannose to an amino acid, was proven.^3^ The delay in this identification was mainly because of the plethora of glycolipids, and peptidoglycan fragments that confounded the early analysis.

Since the seminal demonstration of covalent mannosylation, a few more manno-proteins have been characterized at the molecular level^4–7^ but no one has yet published a concise enumeration of glycoproteins in any given species. There have been a few surveys of mannoprotein content in *M. tuberculosis* (MTB),^8–10^*Streptomyces coelicolor*,^11^*Corynebacterium glutamicum*,^12^*Cellulomonas fimi*,^13^ and one from *Mycobacterium smegmatis*.^14^ Zheng^15^ conducted a survey of *M. bovis*, Bacillus Calmette–Guérin and claimed to have identified more than 700 glycoproteins. However, compared to other surveys, the Zheng data seem at odds with the more typical ≤100 proteins. There are also some surveys from MTB that only “predicted” *O*-glycopeptides^16^ or even *N*-glycans.^17^ It is hard to reconcile the presence of *N*-glycans as they should not be present without the much-studied bacterial oligosaccharyltransferase from the GT-66 family, which is not found in actinobacterial species.^18^ In those latter two surveys, there may be some *O*-mannosylated proteins, but without physical details it is hard to discern whether the results have been validated at the biochemical level. Despite the lack of complete data from any species, there are some common *O*-mannosylated proteins shared between them and some will be discussed below. Key information is missing from all the work thus far, including a molecular mechanism for a function that these glycans serve for the carrier proteins and how that contributes to the normal biology of the bacteria possessing the biochemical machinery for protein mannosylation.

## The protein mannosyl-transferase from glycosyltransferase family GT-39

**Fig. 2:**
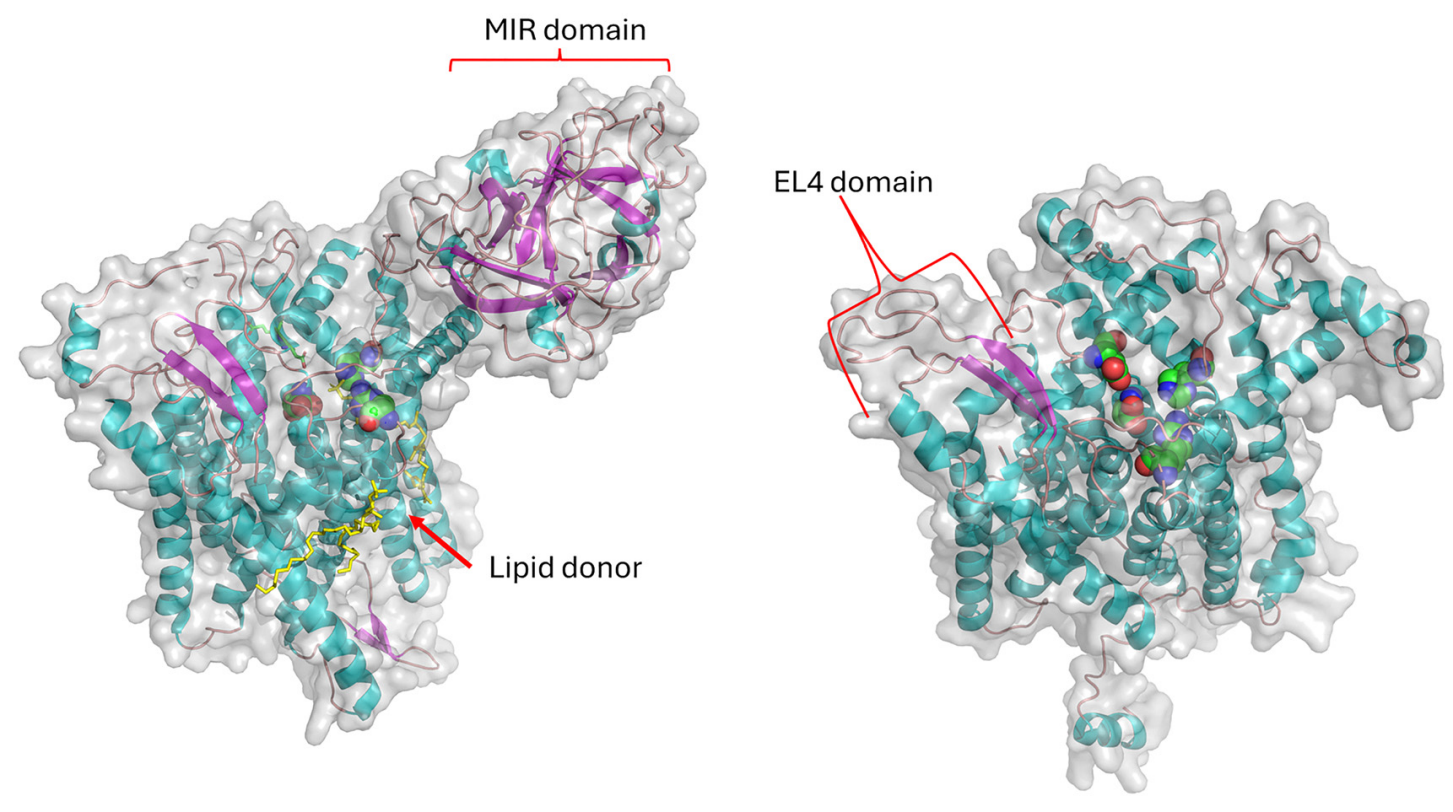
View of the *S. cerevisiae* PMT1 (monomer) structure (left) and the *S. coelicolor* PMT (right) with the conserved catalytic residues highlighted as spheres. In the PMT1 structure the lipid donor is highlighted in yellow. The MIR β-trefoil domain is shown at the top of the yeast PMT1. The EL4 proline recognition domain is shown on the top of the ScoPMT. The yeast structure was derived from PDB 6P25, and *S. coelicolor* structure came from the AlphaFold model AF_Q9RKD3-F1. Figures made with Pymol 3.1.

Work to understand the biochemistry of the GT-39 protein mannosyltransferase started 25 years ago with fungal enzymes. This seminal work is reviewed in.^19^ Subsequent work on the bacterial orthologues showed some similarities with the fungal enzymes (Table 1). Eukaryotes have another more recently described protein *O*-mannosyltransferase, TMTC, in GT-105,^20^ and there are bacterial orthologues but apparently none reported in actinobacteria. The GT-39 enzymes are inverting glycosyltransferases that use a prenol-lipid-linked sugar donor. A direct demonstration of enzymatic activity of the MTB GT-39 protein mannosyltransferase (MtbPMT) was shown in 2002 using cell free assays.^21^ A cryo-EM structure for the *Saccharomyces cerevisiae* PMT1/2 enzyme complex was recently solved.^22^ This has served as a model for the AlphaFold-derived models of the bacterial orthologues. The conservation of the transmembrane helix arrangement of this multi-pass membrane protein can be seen in Fig. 2.

**Table 1:** Percent sequence identity of PMT enzymes from select Actinobacteria^a^ compared to *Saccharomyces cerevisiae* PMT1

PMT source – genome location/uniprot ID	PMT1	Mtb	Sco	Cg	Cfi
*S. cerevisiae* P33775 (PMT1)	**100**	22	26	26	22
*M. tuberculosis Hv1002* (MtbPMT)	22	**100**	39	42	38
*S. coelicolorSco_3154* (ScoPMT)	26	39	**100**	34	43
*C. glutamicum* Cg_1014 (CgPMT)	26	42	34	**100**	31
*C. fimi* Celf_3080 (CfiPMT)	22	38	43	31	**100**

a These GT-39 enzymes are from bacterial species who have had a published survey of mannoproteins conducted, and they are compared to the Saccharomyces enzyme for which there is a cryo-EM based structure.

**Fig. 3:**
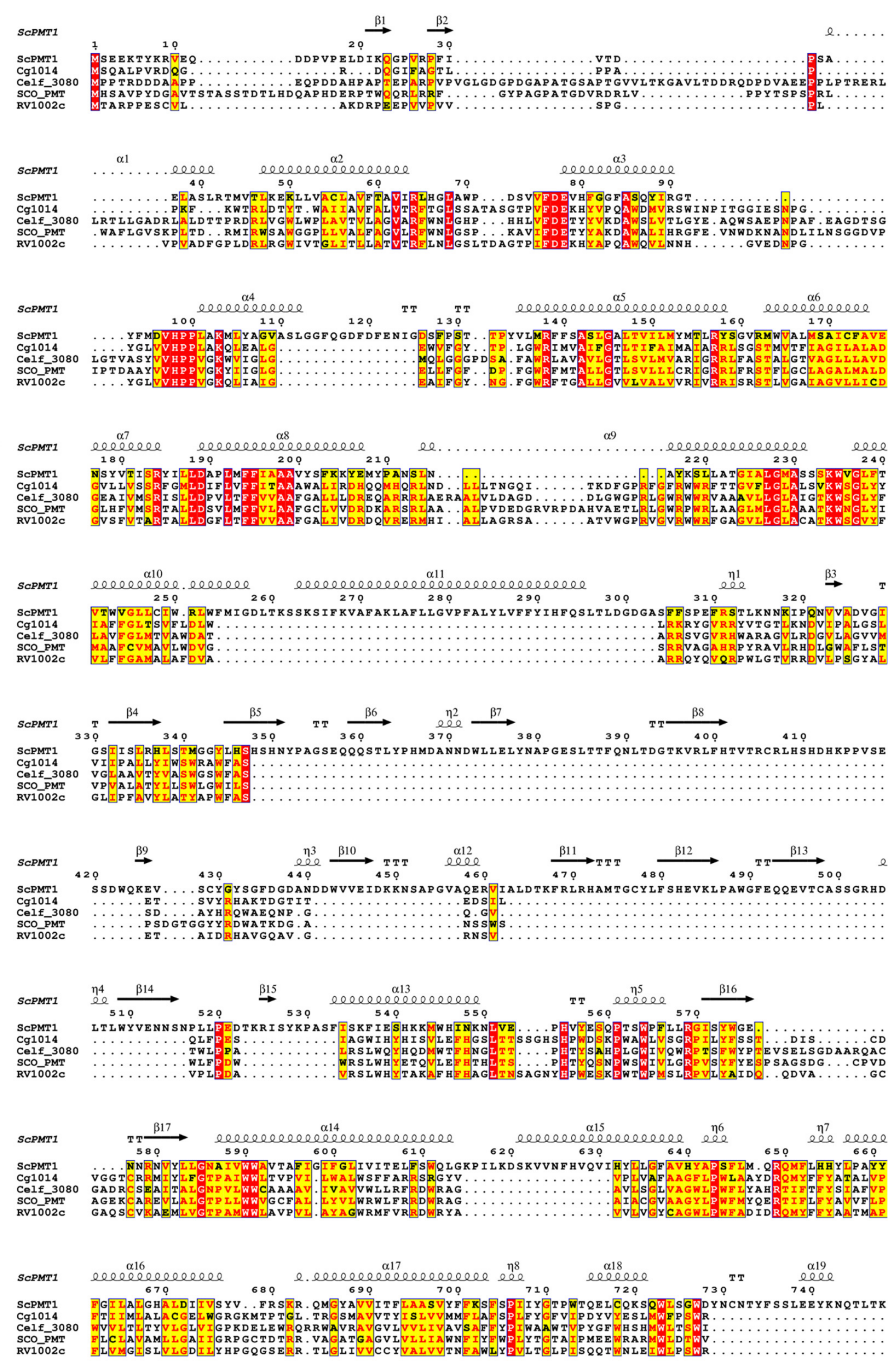
2D sequence alignment against secondary structure elements of the yeast PMT1 structure. The sequence alignment was generated using the T-coffee server.^30^

There are several major differences between the yeast PMT and actinobacterial PMT: (a) the yeast enzyme is firstly a heterodimer, and (b) at the monomer level, differences appear in the loops from the catalytic centre and, where substrate recognition take place; (c) the secondary structure analysis indicates there is an extra helical segment in the *S. cerevisiae* PMT1 sequence (Fig. 3); (d) there is also the interface where the protein–protein interaction for dimer formation takes place, the β-trefoil MIR domain (MIR domains have a β-trefoil fold consisting of six β-hairpins arranged within a pseudo-threefold symmetry); (e) the yeast PMT enzyme monomer is also considerably larger (∼800 residues compared with ∼550 residues for the actinobacterial enzyme). Currently, it is not known if the bacterial enzymes need to form dimers to be functional if so, the bacterial enzymes would form homodimers.

More recently, site-directed mutagenesis of the *S. coelicolor* PMT (ScoPMT) revealed an interesting phenomenon, in that many of the mutants did not produce detectable protein when analysed in western blots from PMT complementation experiments.^23^ A similar problem was seen in work with *C. glutamicum* where the expression of the native protein (CgPMT) expressed *in trans* could not be detected by western blotting analysis, only *via* complementation of the enzyme's activity on reporter proteins.^24^ Two other PMTs were examined in that study, one from *Cellulomonas fimi* (CfiPMT) and one from *Cellulomonas flavigena* (CflaPMT). Neither could be detected directly by western blot analysis. In the work on the *M. smegmatis* and MTB PMT, there was no indication of whether the PMT protein itself could be directly detected in the complemented strains as those strains also used a reporter protein strategy.^25^ The results suggest that the protein's expression and localization are sensitive to subtle changes in structure. It is also likely that the enzyme is present at very low levels, even as a recombinant protein. This lack of detectable overexpression would suggest that regulation may exist at the level of translation which limits how much PMT is made and inserted in the membrane.

Despite the challenges posed by the detection of PMT protein expression, a few key residues were identified in the ScoPMT, which were conserved with the yeast PMT. Site-directed mutagenesis suggested that the amino acids D113, H159, D233, and R510 were part of the active site.^23^ (Fig. 2). In the 2D homology alignment against the yeast PMT1 secondary structure (Fig. 3), some regions of sequence conservation appear in the helical bundles and therefore likely key structural features. While these residues are conserved within this GT-39 enzyme family, it is also worth pointing out that overall, the sequence similarity between the bacterial orthologues is quite low. The highest sequence identity is between ScoPMT and CfiPMT at 43%.

Despite this low overall sequence identity, there was good complementation of the *C. glutamicum* ΔPMT mutantby the CfiPMT even at only 31% protein sequence identity. However, the complementation was not complete, and this suggests distinct substrate specificity of each enzyme, as was observed by Saxena *et al.*^24^ where complementation by orthologues was not always successful. Earlier work with the *S. coelicolor* ΔPMT strain showed that the MtbPMT could not complement the glycosylation of the heterologously expressed MTB APA protein (Rv1860), nor the endogenous PTS protein, which is a Φ31 phage receptor.^26^ This early observation coupled with recent ones suggests that PMTs require precise localization or other protein interactions to be active.

In a 2025 paper by Géraud *et al.*,^27^ the overall topology of the MtbPMT was examined using N- and C-terminal fusion proteins in the surrogate host *M. smegmatis* with the ΔPMT mutation. N-Terminal fusions of the reporter PhoA alkaline phosphatase were not active, whereas the C-terminal fusions were, showing that the C-terminal end is in the periplasmic space. In the same paper, the localization of the PMT was examined with fluorescence reporter fusions – and again the C-terminal end being a highly oxidizing environment, yielded low fluorescence compared to the N-terminal fusions, which were much brighter. This methodology shows that a GT-39 PMT enzyme can be detected in living cells, which means it may be possible to examine protein interactions to further define this complex post-translation event in actinobacteria.

The MtbPMT active site structure was also probed with a series of mutations which expand our understanding of those conserved residues mentioned above in the context of the ScoPMT. There was a newer finding from that study that the EL4 C-terminal domain provides substrate recognition similar to that of eukaryotic WW domain found in proline recognition domains (reviewed in ref. 28). This structural similarity between the EL4 and WW domains reflects the use of high proline content in many MTB glycopeptides.

For *M. tuberculosis* and *M. smegmatis*, a reporter protein approach was used to examine the MtbPMT and MsmegPMT knockouts.^25^ The fasciclin domain protein (UniProt A0R2Q4_MYCS2) was used as a secreted reporter protein. This 27 kDa major supernatant protein, proved to be an excellent protein for analysis, as the N-terminal peptide from aa30-55 contained the three sites of glycosylation. This reporter protein strategy is an exceptional tool to investigate structure-function of the pathway components. It should be noted that this 2013 study also identified the PimE protein as the elongating GT-87 family mannosyltransferase in Mycobacterium where di- and tri-saccharides are the end result. Other actinobacterial species lack a direct orthologue of PimE, but do harbour GT-87 family members which appear to perform the mannose chain elongation.

The consequences of the PMT gene knockouts have been examined n MTB, *M. smegmatis*,^25^*S. coelicolor*,^11^ and *C. glutamicum*.^12,24^ For *C. glutamicum and M. smegmatis*, there was no obvious growth defect, nor were there cell wall defects in the ΔPMT mutant. However, in MTB and *S. coelicolor* there were some notable changes in lab growth and new β-lactam sensitivities. It should be mentioned that in the paper by Keenan *et al.* they state in reference to the ΔPMT strains that “they (*M. smegmatis and C. glutamicum*) are strongly retarded in growth”, however in the reference given for work on *C. glutamicum*^12^ and in our own work,^24^ there was no evidence of a strong growth phenotype in *C. glutamicum* resulting from the PMT gene knockout.

MTB showed a strongly altered growth phenotype, especially on solid media, and a decreased virulence in a mouse model. There was no evidence of cell wall defects based on survival in the presence of chaotropes, which have been found to exacerbate cell wall defects.^29^ The *S. coelicolor* ΔPMT mutant showed an increase in susceptibility to some β-lactam antibiotics which points to peptidoglycan metabolism as a target pathway, and indeed when two glycoproteins involved in peptidoglycan metabolism were inactivated through gene knock out, an increased sensitivity to β-lactams was observed, similar to what was seen for the ΔPMT strain.

## Target proteins of the PMT enzymes

The early reports of actinobacterial glycoproteins were likely dismissed as anomalies, as the existence of glycoproteins in bacteria was not widely recognized until modern mass spectral techniques proved covalent attachment and large-scale sequencing projects, revealing the genetic basis for protein glycosylation now known to be commonplace in the prokaryotic world.^31–34^ Proteins specifically modified with mannose were observed in the 1970s in *M. smegmatis*,^2^ and in that decade they were also observed in secreted cellulases from *Cellulomonas* species.^35^ Characterization of the actual biochemical pathway for this post-translational modification took a longer time, and the breakthrough paper appeared only in 2005, in which the mycobacterial pathway was revealed to be like the eukaryotic GT-39 POMT pathway.^36^ One differentiating feature of the bacterial proteins is that the mannose modification is only 1–3 mannose residues long and has no other sugars as part of the elongation process. Eukaryotic mannoproteins often have longer mannose glycans and in humans these can be capped by other sugars.^1^ Key questions for the bacterial PMT research remain from the work thus far: what are the targets of mannosylation and are these targets limited to only secreted proteins?

The application of proteomics technology has enabled a few surveys of proteins, as noted above. So far, the analysis of these surveys has largely been limited to secreted proteins. Or those found in culture supernatants and this analysis has not addressed how these proteins are represented in the wider actinobacterial community. To understand the broader implications of these proteins in the context of actinobacterial growth and physiology, it is important to look at the types of *O*-mannosylated proteins that have been identified.

### Secreted enzymes

**Fig. 4:**
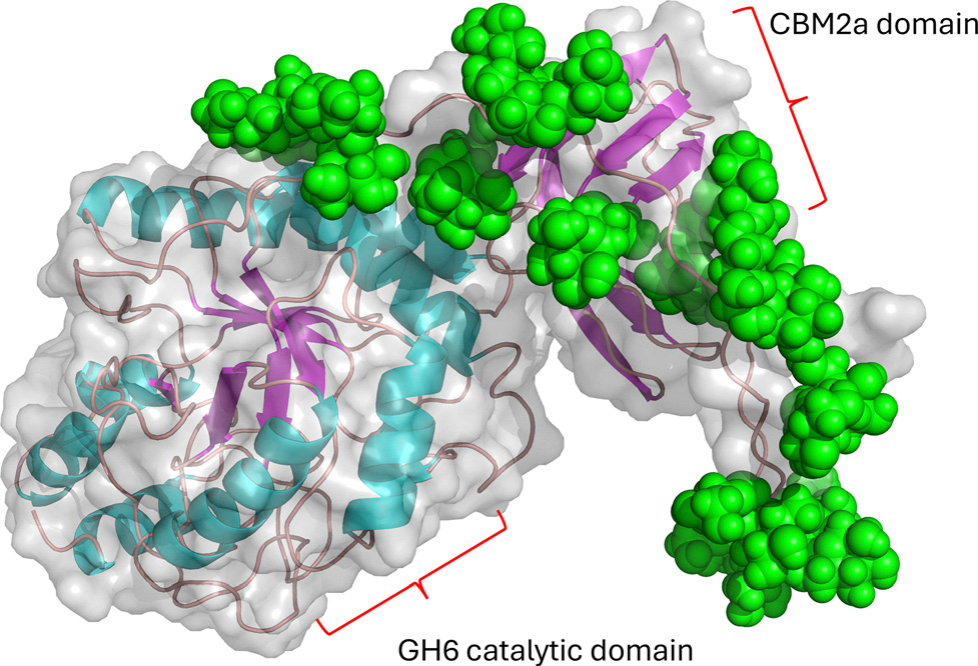
AlphaFold model of Celf_3184 after using Re-Glyco software to display possible mannose modifications.

Many members of the actinobacterial group produce large numbers of secreted glycosylated enzymes, which break down plant cell wall polysaccharides to provide ready access to fermentable sugars.^37–39^ Perhaps the best studied mannosylated example is from *C. fimi*, where the POM initially appeared to be limited to the linker between domains of the GH10 family xylanase or GH6 family cellulase and a CBM2a cellulose-binding module.^40,41^ This domain is a repeating sequence of Pro-Thr and is very heavily glycosylated with up to 32 mannose residues on the Celf_3184 protein (Uniprot F4GZY2_CELFA)^24^ (Fig. 4). Using the Re-Glyco tool^42^ gives us a model of how the glycan would be presented on this unstructured linker. From the model in Fig. 4, it is not hard to imagine that proteases would have reduced access to this linker with the glycan present. A related GH6 family protein from *C. flavigena* Cfla_1896 (Uniprot D5UEY3_CELFN) shows even more glycosylation on a related, but longer linker, with up to 65 mannose residues. Earlier work speculated that the glycosylated linker afforded some protection from proteases.^40^ However, more recent work with other mannosylated linkers on fungal cellulases has suggested a role in actual binding to the cellulosic substrate.^43^

The linker region Celf_3184 = PTTSPTPTPTPTTPTPTPTPTPTPTPTVT, where we assume most if not all T residues are mannosylated. Mannose residues are shown as green spheres. In the related GH6-CMB2 Cfla_1986, the linker is:

TPTPTPTPSVTPSPTPSVTPSPTPSVTPSPTPSVTPSPTPSVTPSPTPSPTVSPTPSPTPSPT, where there must be at least disaccharides on each of the S/T residues to account for the 65 mannose residues.

One interesting functional oddity from the mannosylation of these cellulases is that deleting the PMT gene in *Caldicellulosiruptor bescii* (clearly a GT-39 glycosyltransferase family member Uniprot B9MKU4_CALBD) leads to lack of both protein glycosylation and the secretion of the major cellulase CelA,^44^ which is a cell-wall-anchored polyprotein (Uniprot B9MKU7_CALBD). This protein has five domains: a 3× CBM3, a GH48, and a GH9. These domains are separated by a linker much like what is seen in Cellulomonas endoglucanases, where a repeat of the sequence TPTPTATATP (and slight variants) is the likely site of glycosylation. An important observation is that in *C. besii* it appears that glycosylation is obligately linked to secretion. There is, however, conflicting data showing that the glycosylation is composed of a galactose disaccharide instead of mannose.^45^ However, since the glycosyltransferase mutation made previously is in the GT-39 protein mannosyltransferase family, this galactose moiety seems unlikely, and should be further investigated. There was also the related observation from Saxena *et al.*,^24^ that the *C. fimi* Celf_3184 (CBM2-GH6) endoglucanase reporter protein in the *C. glutamicum* PMT complementation assays was not secreted without functional protein glycosylation. The intrigue arises as the *C. besii* CelA protein uses a Sec secretion leader, while the *C. fimi* Celf_3184 uses a TAT-type secretion leader. This adds a level of complexity to the timing and location of glycan addition if these two modes of secretion require an active PMT for functionality.

**Fig. 5:**
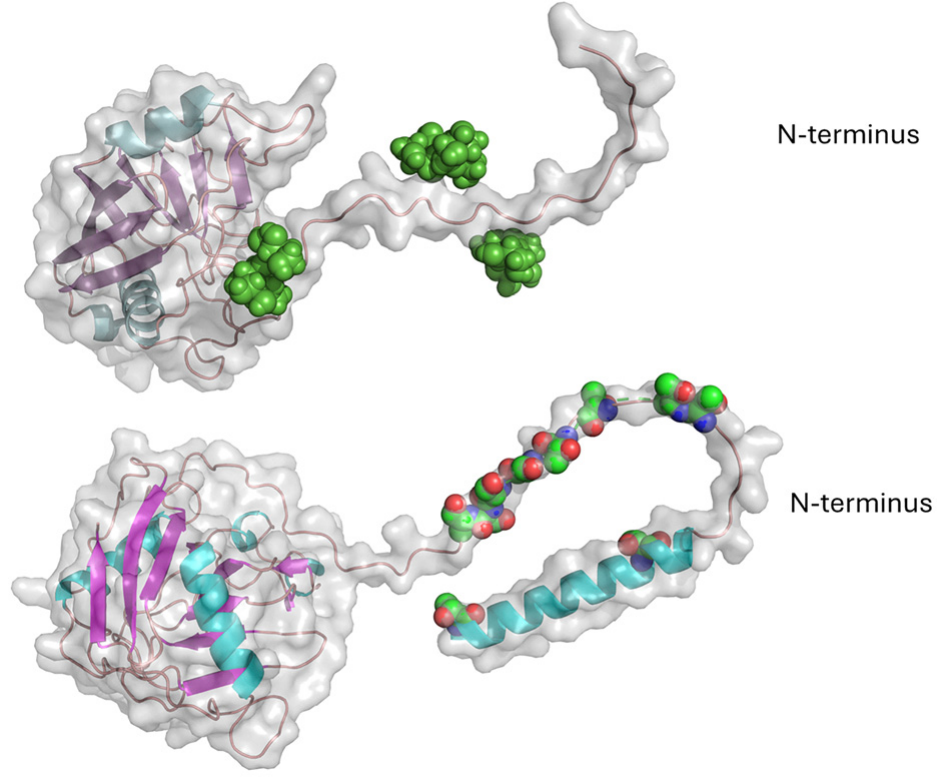
Peptide prolyl isomerases. Upper image: AlphaFold model of Celf_2022 after using Re-Glyco software. Celf_2022 is the *C. fimi* cyclophilin type PPi. The experimentally shown leader sequence of 51 aa residues has been removed from this model. Lower image: Rv2582/PPiB from *M. tuberculosis*. The spheres are the possible Thr/Ser sites of mannosylation in the N-terminal segment after removing the first 46 aa residues from the model in analogy to the truncation of the Celf_2022 model.

The secreted and mannosylated glycoside hydrolases are certainly common in many actinobacteria. Notably, *Mycobacterium* and *Corynebacterium* lack these kinds of enzymes. One protein that is common between the mannoproteome-surveyed organisms is a class of secreted proteins annotated as a peptide prolyl isomerase (PPi). These proteins have been identified in MTB, *C. fimi*, *S. coelicolor* and *C. glutamicum*, and shown to be glycosylated in three of them (Table 2). These proteins have between 34 and 45% sequence identity, and are all characterized as having extraordinarily long leader sequences that are not SEC/TAT or lipoprotein but “other” based on the SignalP algorithm. The leader sequence for the Celf_2022 protein was shown experimentally to be 53 amino acids long and the glycosylation occurred at a site immediately following the cleaved leader sequence (Fig. 5). The site of glycosylation on the other PPi orthologues is unknown. The *C. fimi* PPi protein is secreted with and without glycosylation^24^ which begs the question, what is the purpose of the modification?

**Table 2:** Secreted glycoproteins annotated as peptide prolyl isomerases

Organism	Gene/protein ID	Uniprot ID	Ref.
*M. tuberculosis*	Rv2582/PPiB	P9WHW1 cyclophilin type	8
*S. coelicolor*	SCO1510	Q9KXP0 cyclophilin type	Not identified as a glycoprotein
*C. fimi*	Celf_2022	F4H0A5 cyclophilin type	13
*C. fimi*	Celf_3689	F4H4K1 FKBP type	13
*C. glutamicum* a	WP_011014511.1	Cyclophilin type	NA

a Included based on homology with the other cyclophilin orthologues.

The MTB PPiB is an essential protein for MTB,^46^ as well as having both prolyl-isomerase and protein chaperone activity through its ability to stabilize proteins.^47^ This protein is found in the secretome and in membrane fractions of MTB. It has generated an immune response in TB patients and can also modulate the immune response by altering cytokine profiles.^48^ Functional protein can be made in *Escherichia coli*, so mannosylation is not required for the chaperone function. Although in these studies a direct comparison of glycosylated and non-glycosylated protein was not performed. An investigation of other orthologues, which are expressed as mannosylated proteins, perhaps with PPi/chaperone functional assays, may provide more insight into glycosylation's role for these small, truly secreted proteins.

The function of PPi proteins other than MTB PPiB has not been experimentally verified, but we know that the *C. besii* CelA protein also relies on a functional PPi for efficient export to the cell surface, although it should be noted that this PPi appears to be membrane-anchored as opposed to truly secreted. It would be interesting to know if the *C. besii* PPi is also a glycoprotein and if the PPi/target protein interaction depends on protein glycosylation.

## Proteins involved in peptidoglycan metabolism

One interesting group of common proteins from the mannosylation glycoproteome surveys are those involved in peptidoglycan metabolism. They have consistently shown up in the various glycoproteomics experiments that we are reviewing here (Table 3). As the metabolism of peptidoglycan requires proteins to work together and there are known protein complexes, the presence of glycosylation on some members of this group makes it tempting to speculate that a protein–carbohydrate interaction plays a role in the formation of these complexes.

**Table 3:** Proteins involved in peptidoglycan metabolism

Organism	Penicillin binding protein	Pasta domain kinase	d,d/l,d endopeptidases
*M. tuberculosis*	PonA2 – PBP1a-like RV3682^a^	Rv0014c	ND
		Uniprot-P9WI81	
*M. smegmatis*	PonA2 like	ND	ND
	Uniprot-A0R5I3_MYCS2		
*S. coelicolor*	SCO4013	SCO3848	SCO4394, SCO4847
	Uniprot-Q9ADP3	Uniprot-Q9XA16	
*C. fimi*	Celf_0189	Celf_0029	ND
	PBP2-like	Uniprot-F4H3V7_CELFA	
	Uniprot-F4H5L1_CELFA		
*C. glutamicum* b	PBP1-like	Uniprot-Q8NU98	Uniprot-Q8NSJ1_CORGL
	Uniprot-Q8NLF6_CORGL		

a The genome location and/or the uniprot ID is given for each protein.

b This is included simply because *C. glutamicum* protein is homologous to the other orthologues.

## Penicillin-binding proteins

It is quite striking that several of these conserved actinobacterial proteins are involved in peptidoglycan metabolism. As mentioned above, there are at least two mannoproteins in *S*. *coelicolor* which are d,d/d,l-transpeptidases (Class C PBP). When these particular mannoproteins are deleted, these cells become more susceptible to cell-wall-active β-lactams. We identified and published a penicillin-binding protein from *Cellulomonas* and showed that the glycopeptide was part of the endo-peptidase domain of this PBP2-like protein (Class B PBP). The other actinobacteria we have been discussing here have a PBP1-like protein (Class A PBP), which is a two-domain protein comprised of the GT-51 glycosyltransferase domain as well as an endo-peptidase domain.

**Fig. 6:**
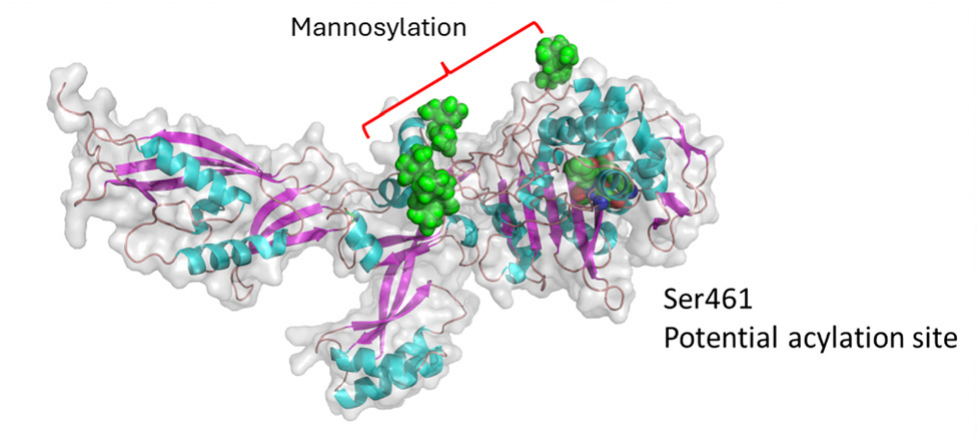
AlphaFold model of Celf_0189 after using Re-glyco software. Celf_0189 is a *C. fimi* PBP2-like protein. The potential site of acylation by a β-lactam is Ser461 and is shown in a spheres representation.

**Fig. 7:**
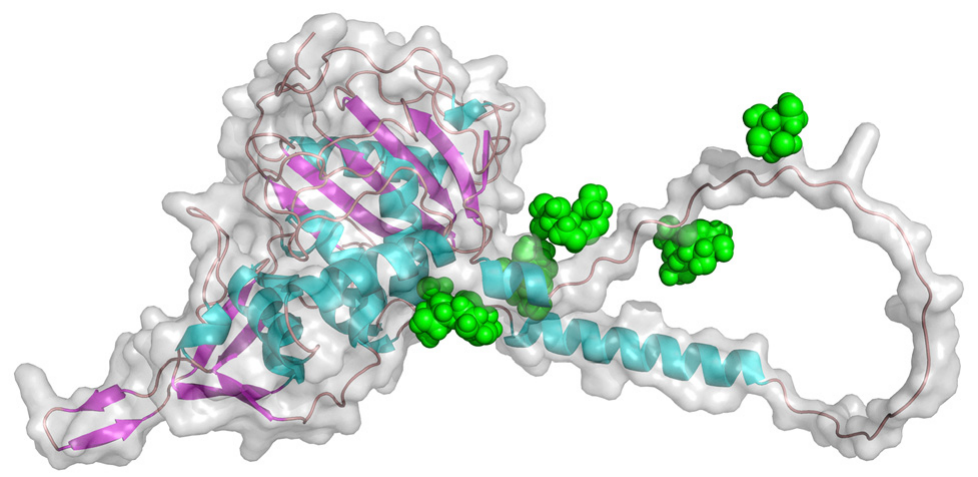
AlphaFold model of SCO4847 after using Re-Glyco software. SCO4847 is a Class C PBP which is annotated as an endopeptidase.

From the available data it does not appear that all the PBPs are glycoproteins, but rather that only select members are mannosylated. Further, they are not all in the same class of PBP. In MTB and *M. smegmatis*, PonA2 is Class A PBP1-like protein, in *C. fimi*, Celf_0189 is a Class B PBP2-like protein, and in *S. coelicolor*, there are two Class C PBPs. In Fig. 6, the AlphaFold model of Celf_0189 is shown as its re-glycosylated form based on published glycopeptide data. In this case the glycosylation appears to be in a place where it could influence protein–protein interactions. Fig. 7 shows the re-glycosylation model of SCO4847. As with the other models, the glycosylation appears to be on an unstructured loop, which is common for *O*-glycosylation. It is tempting to speculate that the combination of mobility of the loop and the presence of the mannose helps direct this protein to a binding partner(s) or to interact with the peptidoglycan substrate.

## PASTA domain kinases

**Fig. 8:**
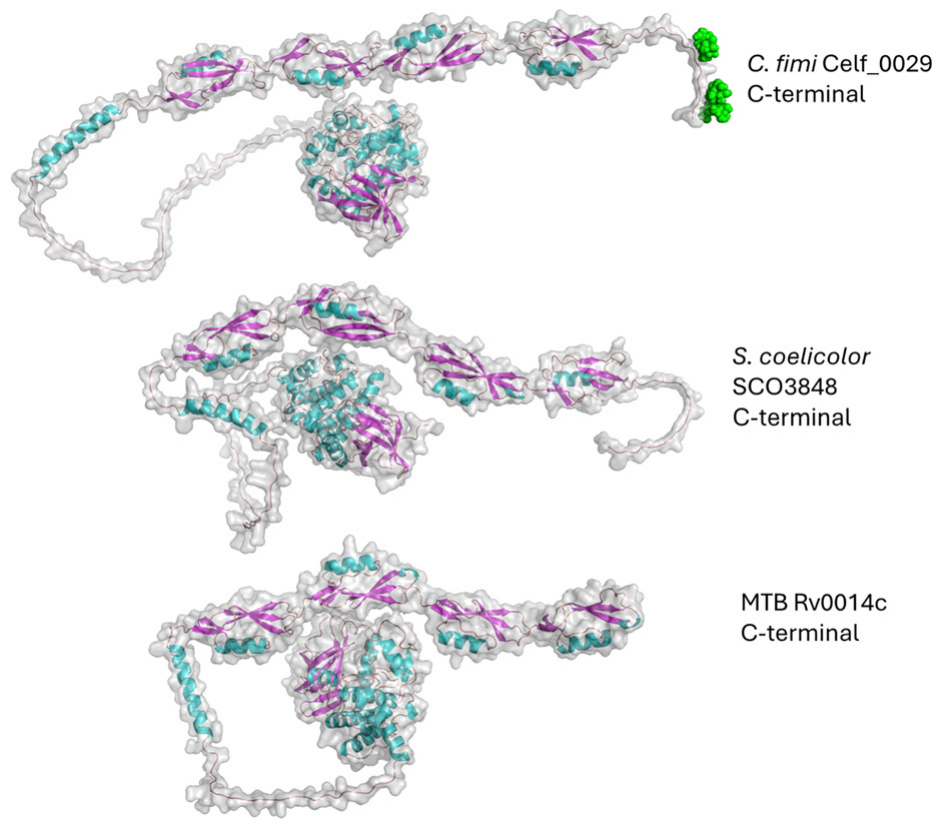
AlphaFold models for PASTA domain kinases from *C. fimi*, *S. coelicolor*, and MTB.

The next group of proteins from the class of peptidoglycan active proteins is that of the PASTA domain kinases. These kinases are Ser/Thr active kinases, and PknB has been shown to be essential in MTB.^49^ A PASTA domain kinase has been shown to be glycosylated in MTB, *S. coelicolor*, and *C. fimi* (Fig. 8).^8,11,13^ To regulate cell growth and cell division in MTB, the PASTA domain kinases have been involved in binding muropeptides and in a proposed model suggests PknB is targeted to the sites of peptidoglycan turnover.^50^ In the case of the *C. fimi* PASTA domain kinase, the glycosylation is at the extreme end of the last PASTA domain. However, we do not have the glycopeptide information for the MTB enzyme.

We know from work with MTB PknB that all the PASTA domains are required for proper activity, and that they are responsible for localization within the cell. PknB is also an essential enzyme in MTB, as is the orthologue in *C. glutamicum*.^51^ An interesting possibility is that the glycosylation on the PknB may play a role in interacting with PknB's various substrate proteins, or with muropeptides which help regulate its activity, and that this is why the MTB glycosylation mutant has a growth defect. This could also be why *S. coelicolor* has a growth defect. However, that does not appear to be the case for *C. glutamicum*, as the ΔPMT mutant has no noticeable growth defect in a lab setting.

These enzymes are characterized by the presence of four repeated PASTA domains on the C-terminal end of the structure. The *C. fimi* protein has been through the Re-Glyco software to show where the mannose glycan (green spheres) is found, based on the reported glycopeptide data.

Looking at the models, it is easy to see the similarities between the *C. fimi* and *S. coelicolor* orthologues, where they both have a prominent tail on the C-terminal end. Because the MTB version lacks the C-terminal tail, it is not easy to see where the mannose could be on that version. Obviously, knowing where the glycan is on these other PASTA domain kinases would help with understanding why they carry glycans.

## GH3 glycoside hydrolases for peptidoglycan recycling

The MTB protein LpqI is annotated as a glycosidase from the GH3 family (Table 4). This protein was identified in the glycoproteomics survey from Gonzalez-Zamorano *et al.*^8^ The MTB protein has been extensively characterized, including a crystal structure, but from a truncated version expressed in *E. coli*.^52^ This protein cleaves the GlcNAc–MurNAc glycan backbone of the peptidoglycan fragments, thereby helping to recycle the peptidoglycan. An orthologue has been cloned from *C. fimi*^53^ but no investigation of its possible glycoprotein status was investigated. The *C. fimi* protein has also been shown to prefer phosphorylated substrates,^54^ which would be unusual if the only purpose was GlcNAc–MurNac hydrolysis. The *C. glutamicum* protein has also been partially characterized,^55^ and it was shown to cleave simple *para*-nitrophenyl-*N*-acetylglucosamine. Like MTB, the *C. glutamicum* protein is an orphan GH3 family glycoside hydrolase in it's parent organism, like LpqI in MTB, that lacks the ability to hydrolyze external glycans. Confirmation of the other members of this group as mannoproteins is needed, as well as showing that they indeed cleave the GlcNAc–MurNAc glycosidic bond. What still needs to be examined is the role that POM plays for this membrane-associated hydrolase.

**Table 4:** GH3 NagZ or LpqI orthologues

Organism	Gene/protein ID	Ref.
*M. tuberculosis*	LpqI, Rv0237	8
	L7N6B0	
*S. coelicolor*	Q93RU1_STRCO	Not yet identified as a glycoprotein
*C. fimi*	Q7WUL3·NAG3_CELFI	Not yet identified as a glycoprotein
*C. glutamicum*	Q8NLT5_CORGL	Not yet identified as a glycoprotein

## Protein *O*-mannosylation beyond discovery research

Recombinant expression of peptide-fusions and various mycobacterial proteins has been used to determine site preferences for the PMT and thus showed the power of expression in surrogate hosts. An early exploration of glycosylation sites using the algorithm Net-*O*-Glyc showed that eight of 11 predicted sites of MTB proteins could be glycosylated in *M. smegmatis*.^56^ This same approach was employed in the seminal 2005 publication by VanderVen *et al.*, who used a non-glycoprotein from the Ag85 complex fused to an APA protein glycopeptide to investigate the secretion of mycobacterial glycoproteins.^36^ Other early observations about POM were that certain surface proteins from MTB and *M. bovis* BCG that carried this modification might be vaccine targets. These immunodominant proteins are Ala-Pro rich antigen, APA (Rv1860); MPB83 (BQ2027_MB2898)/MPB70 (BQ2027_MB2900); and PstS1 (Rv0934). These proteins have been shown to act as adhesins and may provide a pathway to being taken up into macrophages where they hide from the immune system and proliferate.^57^ They are also subject to upregulation under phosphate starvation, which leads to higher uptake by macrophages.^58^ Since they were discovered and their immunodominance observed, several papers have documented their production in alternate expression hosts.

One of the first papers to look at recombinant glycosylated APA proteins describes the use of the surrogate host *S. lividans* to produce the so-called 45 kDa and 47 kDa antigens (APA, Rv1860) as mannoproteins.^59^ These glycoproteins had been shown to stimulate T-cells,^60^ and immune serum from TB patients only reacted to the glycosylated forms of the proteins. This use of the surrogate host showed that native glycosylation was possible even with the heterologous protein. Optimizing the expression of APA in *S. lividans* showed that it is important to be careful when scaling up to maintain yields and protein quality.^61^ This also suggests that regulating recombinant protein mannosylation is coupled to as-yet unknown metabolic cues in growing cells. It should also be noted that *S. lividans* was used more than a decade earlier to produce a mannosylated a “PT” sequence repeat linker in a *C. fimi* GH-10 family xylanase like the GH6 family endoglucanase noted earlier in this paper, and that the glycosylation was again true to the native pattern previously observed.^41^ This shows that *S. lividans* is very versatile, as it can express both mycobacterial and *Cellulomonas* glycoproteins with accurate glycosylation.

The use of surrogate hosts has permitted a deeper understanding of the biochemistry of APA proteins as they interact with mammalian receptors. An interesting study from 2007 showed that the human pulmonary C-type lectin surfactant protein A required mannosylation on the 45/47 kDa APA and may be an adhesin that permits host cells to colonize by taking advantage of this innate immune receptor.^62^ This study again made use of the *M. smegmatis* expression system to produce enough proteins for the receptor- and antibody-binding studies. *M. smegmatis* as a closer relative to MTB is excellent for providing authentic expression of these glycoproteins

A new surrogate host, *Rhodococcus erythropoli*s, was introduced to express not only the MTB APA proteins but also the Pts1 and LprG proteins.^63^ The MTB Pts1 protein is part of the phosphate transport system and an orthologue of the first *S. coelicolor* glycoprotein to be described in detail.^5^ Saxena *et al.* noted that Cellulomonas also had a glycosylated orthologue of this protein.^13^ In MTB, Pts1 is the 38 kDa antigen and is an immunodominant antigen^64^ like the APA and LprG proteins. While this expression system did produce all three proteins with mannosylation, the yields were quite low, and it appears that the system would require a great deal of optimization before it could be as valuable as *S. lividans*.

More recently, the quest for vaccine candidate validation for APA has been examined in greater detail with protein produced with and without mannosylation.^65^ For this paper, they used proteins produced in *E. coli* and *S. lividans* to provide the antigens. Immunization of mice revealed that the two proteins induce different cytokine responses – and that the mannosylated protein could stimulate a proliferative response from T-cells. The complex response of the cytokines and immune cells suggests that we still don’t know the mechanism by which the mannosylated proteins stimulate the response, even though we know some of the receptors that interact directly with the APA proteins.

The immunodominant mannosylated antigens and their possible role in host colonization have given rise to the idea of POM as a target for antibiotic development. From a chemical biology perspective, more work would have to be done to determine if POM is an ideal target for an antibiotic; other glycans in MTB have been studied much more closely to determine their suitability as target for antibiotics.^66,67^ Recent research from Géraud *et al.*^68^ has shown a reporter protein-based assay for the assessment of potential inhibitors. While the screen had only a few compounds, it demonstrated that an *in cellulo*-based assay for POM/PMT activity is feasible in Mycobacteria. A note of caution was issued regarding compound uptake however, as Mycobacteria have a very hydrophobic cell wall, which means that hydrophobic compounds are likely to have limited uptake through the cell-wall barrier.

## Conclusions

The literature on protein mannosylation in actinobacteria has been slowly increasing over the last 50 years. The predominant focus has been on the MTB cell-wall antigen proteins, with a secondary interest in secreted glycoside hydrolases. While structural details of the protein targets of POM are scarce, we have presented here a snapshot of models for discussion using modern software tools that were previously unavailable. The vast improvements in predicting protein structure by AlphaFold coupled with the ability to use web-based molecular dynamics allows us to “visualize” the products of POM. It is striking that we now have models which allow us to test structure-function in these proteins, a case in point being the PMT work from Géraud *et al.*,^27^ where site-directed mutagenesis based on the AlphaFold model has provided new insight into the EL4 domain on the MTB PMT.

The use of re-glycosylated protein models also allows us to think about how these glycans can be used to interact with other proteins. This is especially true in the case of the group of proteins involved in peptidoglycan metabolism. Peptidoglycan-active proteins are known to form complexes, and glycan–protein interactions are common in other cellular contexts. This leads to a discussion of how the lack of POM leads to growth defects. The major defect appears to be cell-wall-related in both MTB and *S. coelicolor*, which certainly fits with the idea of some defect in peptidoglycan metabolism. While the lack of POM in MTB and *S. coelicolor* leads to some biological consequence, this is not the case for *C. glutamicum* and *M. smegmatis.* This sets up the next area for follow-up research in *C. glutamicum* and *M. smegmatis*: under what conditions would lack of POM have biological consequences?

MTB's use of POM to provide a means to enter macrophages (along with other glycans) continues to present an interesting intervention point for anti-infective strategies. The fact that we are still learning about those adhesin/antigenic proteins that were discovered decades ago makes it clear that we have more to discover about the functional role of POM for MTB. Without a solid conclusion about POM's biological role in MTB outside of infectivity, what remains to be discovered in the nearly 4000 other species of bacteria that also harbour a GT-39 family PMT? Questions about the role of the glycosylation will need to be addressed with existing genetic manipulations, but finding a phenotype for lab-grown strains will require consideration about different growth conditions, and perhaps a variety of stressors to assist in finding the link between POM and Actinobacterial growth and physiology. Our hope is that this review will spark some interest in developing new ways to probe this enigmatic post-translational modification.

## Conflicts of interest

There are no conflicts to declare.

## Data Availability

There is no original data in this manuscript to make available.
